# Radiographic assessment of the skeletons of Dolly and other clones finds no abnormal osteoarthritis

**DOI:** 10.1038/s41598-017-15902-8

**Published:** 2017-11-23

**Authors:** S. A. Corr, D. S. Gardner, S. Langley-Hobbs, M. G. Ness, A. C. Kitchener, K. D. Sinclair

**Affiliations:** 1 Schools of Veterinary Medicine and Science and Biosciences, University of Nottingham, Leicestershire, LE12 5RD UK; 2 School of Veterinary Medicine, College of Medical, Veterinary and Life Sciences, University of Glasgow, Glasgow, G61 1QH UK; 3 University of Bristol, Langford House, Langford, Bristol, BS40 5DU UK; 4 Longframlington, Northumberland, UK; 5 Department of Natural Sciences, National Museums Scotland, Chambers Street, Edinburgh, EH1 1JF UK

## Abstract

Our recent report detailing the health status of cloned sheep concluded that the animals had aged normally. This is in stark contrast to reports on Dolly (first animal cloned from adult cells) whose diagnoses of osteoarthritis (OA) at 5½ years of age led to considerable scientific concern and media debate over the possibility of early-onset age-related diseases in cloned animals. Our study included four 8-year old ewes derived from the cell line that gave rise to Dolly, yet none of our aged sheep showed clinical signs of OA, and they had radiographic evidence of only mild or, in one case, moderate OA. Given that the only formal record of OA in Dolly is a brief mention of a single joint in a conference abstract, this led us to question whether the original concerns about Dolly’s OA were justified. As none of the original clinical or radiographic records were preserved, we undertook radiographic examination of the skeletons of Dolly and her contemporary clones. We report a prevalence and distribution of radiographic-OA similar to that observed in naturally conceived sheep, and our healthy aged cloned sheep. We conclude that the original concerns that cloning had caused early-onset OA in Dolly were unfounded.

## Introduction

The conference abstract by Rhind *et al*.^1^ reported that Dolly^2^ had OA of the left stifle (knee) at 5½ years of age. Radiographs of her right stifle were reportedly normal at that stage. In the absence of the original records, we undertook a detailed radiographic examination of the skeletons of Dolly, along with Bonnie (her naturally conceived daughter), and Megan and Morag (the first two animals to be cloned from differentiated cells^3^) (Supplementary Table 1). These skeletons are in the collections of National Museums Scotland in Edinburgh.

Assessment of the radiographs showed that, as would be expected, the radiographic-OA (rOA) was more severe and affected a greater number of joints in the two older sheep (Bonnie and Megan) compared to Dolly (Table 1). While Bonnie and Megan had evidence of rOA in the majority of their joints, Dolly had no rOA in her shoulder, carpal or hock joints when she was euthanized at 6 years 8 months. Overall, the distribution of rOA was similar to that described in the 7–9 year old cloned sheep by Sinclair *et al*.^4^, affecting mainly the elbows and stifles, with the elbows being consistently worst affected. Morag, a clone of Megan, showed minimal rOA when euthanized at 4½ years. Images of selected bones of Dolly and Morag are shown in Supplementary Fig. 1 and 2.

**Table1:** Scoring of osteoarthritis in Dolly^2^, her naturally-conceived daughter (Bonnie) and two predecessor cloned sheep (i.e. Megan and Morag^3^).

**SHEEP (Age)**		**Hip**				**Stifle**				**Hock**				**Shoulder**				**Elbow**				**Carpus**				**Total (median)** ^**‡**^
		^†^ **a**	**b**	**c**	**sub-total**	**a**	**b**	**c**	**sub-total**	**a**	**b**	**c**	**sub-total**	**a**	**b**	**c**	**sub-total**	**a**	**b**	**c**	**sub-total**	**a**	**b**	**c**	**sub-total**	
DOLLY (6 y 8 m)	**Right**	1	2	2	**5**	1	1	1	**3**	0	0	0	**0**	0	0	0	**0**	2	2	1	**5**	0	0	0	**0**	**13 (0**.**5)**
	**Left**	1	1	1	**3**	2	2	2	**6**	0	0	0	**0**	0	0	0	**0**	3	3	3	**9**	0	0	0	**0**	**18 (0**.**5)**
BONNIE (9 y 11 m)	**Right**	0	0	1	**1**	1	1	1	**3**	2	2	2	**6**	1	1	0	**2**	3	3	2	**8**	1	1	1	**3**	**23 (1**.**0)**
	**Left**	1	1	2	**4**	1	1	1	**3**	3	3	2	**8**	1	1	0	**2**	3	3	2	**8**	1	1	1	**3**	**28 (1**.**0)**
MEGAN (13 y 6 m)	**Right**	1	1	0	**2**	3	3	1	**7**	0	0	1	**1**	0	0	0	**0**	2	3	1	**6**	1	1	0	**2**	**18 (1**.**0)**
	**Left**	0	0	0	**0**	3	3	3	**9**	0	0	1	**1**	0	0	0	**0**	3	3	3	**9**	0	1	0	**1**	**20 (0**.**0)**
MORAG (4 y 8 m)	**Right**	0	0	1	**1**	0	0	0	**0**	0	0	0	**0**	0	0	0	**0**	1	1	0	**2**	0	0	0	**0**	**3 (0**.**0)**
	**Left**	0	1	0	**1**	0	0	0	**0**	0	0	0	**0**	0	0	1	**1**	0	0	0	**0**	0	0	0	**0**	**2 (0**.**0)**
Total (median)^‡^					**17 (1**.**0)**				**31 (1**.**0)**				**16 (0**.**0)**				**5 (0**.**0)**				**47 (2**.**0)**				**9 (0**.**0)**	

^†^a,b,c - Independent scores for evidence of OA by three veterinary orthopaedic specialists.

^‡^Medians for each row are for independent scores across all six joints. Medians for each column are for independent scores of each joint across all four sheep.

Similarly, in the study by Sinclair *et al*.^4^, only one Finn-Dorset (Dolly) clone had widespread moderate rOA and 11/13 cloned sheep had either no or mild rOA in their joints. All were asymptomatic. Although Dolly was lame, studies reporting the prevalence of OA in naturally-conceived sheep suggest clinical OA is not unusual^5^.

It is well established that osteoarthritis can develop as a result of a number of recognised genetic and environmental risk factors. For example, variants in both nuclear and mitochondrial DNA are associated with OA in humans^6^. Although Dolly and the four Finn-Dorset clones reported by Sinclair *et al*.^4^ shared the same nuclear DNA, they will have differed in their mitochondrial DNA. It is not known if these genetic differences could explain some of the variance in rOA between the clones. Additional well-established risk factors for developing OA include age^7^, hence the concerns over Dolly’s diagnosis whilst still relatively young. However *post-mortem* studies of clinically normal, naturally-conceived sheep have identified pathological changes in 66% of stifles of animals aged 6 months to 11 years, with the severity of the lesions increasing with age^8^. This is reflected in our study where Morag had significantly less rOA than Megan, her genomic copy, who was 9 years older. Osteoarthritis can also be initiated by trauma, with the stifle and elbow joints most commonly affected in sheep^9^. Other risk factors for OA include obesity^10^ and pregnancy independent of body-mass index^11^. Although no record remains of Dolly’s body weight, she is known to have produced six lambs, including Bonnie, whilst the four ‘Dolly’ clones reported by Sinclair *et al*.^4^ were not bred.

Any of these risk factors could account for Dolly’s OA, and the different expression of clinical and/or radiographic OA between individuals, irrespective of whether an animal had been cloned or conceived naturally.

Our results should be interpreted within the context of several limitations. Firstly, OA is a disease of the entire joint, including the joint capsule, synovial lining and articular cartilage, but only the bones of Dolly, Bonnie, Megan and Morag remain for examination. Secondly, radiographic changes indicative of OA do not necessarily correlate with the extent of clinical disease^12^, and no clinical information is available on the musculoskeletal health of Bonnie, Megan or Morag.

Nonetheless, we conclude that the prevalence and distribution of rOA in Dolly and her contemporary clones is no different to that observed in naturally conceived sheep, and in the healthy aged cloned sheep described by Sinclair *et al*.^4^. Concerns raised over a direct link between Dolly’s OA and cloning were therefore unfounded.

## Methods

### Radiographs

These were taken of the skeletons of Dolly, Megan, Morag and Bonnie (Supplementary Table 1) held within the collections of National Museums Scotland in Edinburgh. No live-animal assessments were undertaken. Radiographs were obtained of all major limb bones in the right and left forelimbs (scapula, humerus, radius, ulna and metacarpals) and hind limbs (pelvis, femur, tibia, fibula and metatarsals) from all four sheep. Images were obtained using a portable Tru-DR Digital Radiography System, with laptop (R8–51D45) and plate (50-C907–08B; BCF Technology Ltd, Strathclyde, UK). Digital images were subsequently converted to jpegs for analysis using OsiriX Lite (http://www.osirix-viewer.com).

### Analyses

Images were anonymized and independently scored for evidence of OA by three veterinary orthopaedic specialists (indicated in Table 1 as a, b, c) using the modified Kellgren and Lawrence scale as used by Sinclair *et al*.^4^. Presence of OA was scored on a semi-quantitative, categorical scale based on the presence or absence of osteophytes and bone remodelling: 0, no evidence of osteophytes or bone remodelling; 1, mild osteophytosis; 2, moderate osteophytosis and minor bone remodelling and 3, severe osteophytosis with definite bone remodelling. Assessment of joint space width, used clinically on live specimens as an indicator of OA, was not appropriate for use on skeletons. Proximal and distal ends of individual bones were individually scored to produce an average score for each joint. Analysis of the overall degree of agreement/consensus between the three clinicians interpreting radiographs (i.e. inter-rater variability) used Kendall’s coefficient of concordance where a score of 0 = no agreement and 1 = perfect agreement between raters. Two images of six joints from each of four sheep were assessed (48 images in total). All data were analyzed using Genstat v16 (VSNi, Rothampsted, UK). Statistical significance was considered at P < 0.05. Agreement between clinicians assessing radiographs was very strong (Kendall’s Coefficient = 0.86, χ^2^ = 122, P < 0.001).

### Data availability

The radiographs that support the findings of this study are available from the corresponding authors (SAC or KDS) or National Museums of Scotland (ACK) on reasonable request.

## Electronic supplementary material

Supplementary information
