# Supplementary material for: Radiographic assessment of the skeletons of Dolly and other clones finds no abnormal osteoarthritis
Source: Sci Rep. 2017 Nov 23;7:15685. doi: 10.1038/s41598-017-15902-8 (PMC5701071; doi:10.1038/s41598-017-15902-8)
Supplement: Supplementary file 1 — Supplementary information [file 41598_2017_15902_MOESM1_ESM.docx]

**Radiographic assessment of the skeletons of Dolly and other clones finds no abnormal osteoarthritis**

S.A. Corr^1,2*^, D.S. Gardner^1^, S. Langley-Hobbs^3^, M.G. Ness^4^, A.C. Kitchener^5^ and K.D. Sinclair^1*^

^1^Schools of Veterinary Medicine and Science and Biosciences, University of Nottingham, Leicestershire, LE12 5RD, UK; ^2^School of Veterinary Medicine, College of Medical, Veterinary and Life Sciences, University of Glasgow, Glasgow, G61 1QH, UK; ^3^University of Bristol, Langford House, Langford, Bristol, BS40 5DU, UK; ^4^Longframlington, Northumberland, UK; ^5^Department of Natural Sciences, National Museum of Scotland, Chambers Street, Edinburgh, EH1 1JF, UK.

*Correspondence: [sandra.corr@glasgow.ac.uk](mailto:sandra.corr@glasgow.ac.uk) or [kevin.sinclair@nottingham.ac.uk](mailto:kevin.sinclair@nottingham.ac.uk)

**Supplementary Figure 1.** Images of mediolateral radiographs showing: (a) Dolly’s left distal femur, (b) Morag’s left distal femur, (c) Dolly’s left proximal tibia, and (d) Morag’s left proximal tibia. White arrows show osteophytes/areas of bone remodelling. The circular hole in the center of (c) is an artefact, created *post-mortem*.

The individual scores for the distal femur and proximal tibia were averaged to create a median score for each stifle joint: Dolly’s left stifle was scored as 2, Morag’s as O.


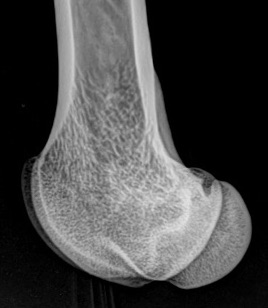


b


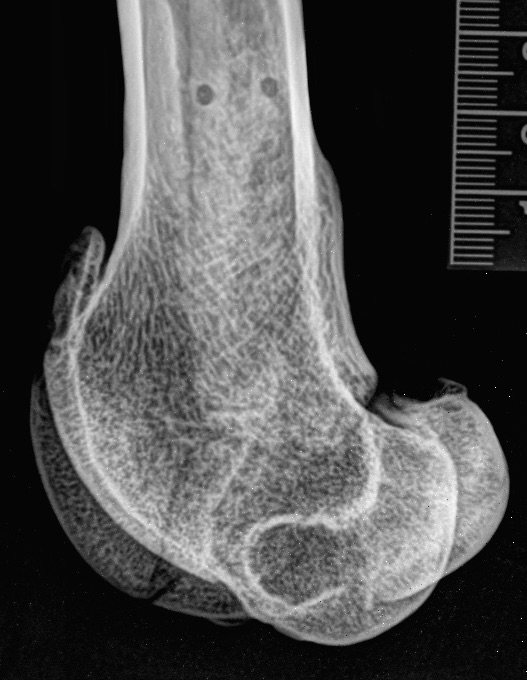


a


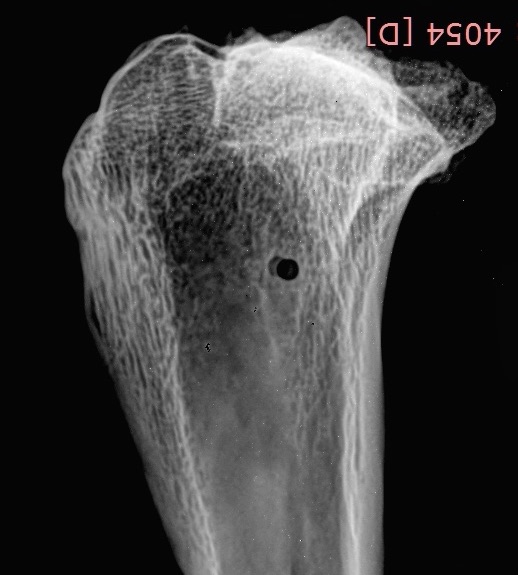


c


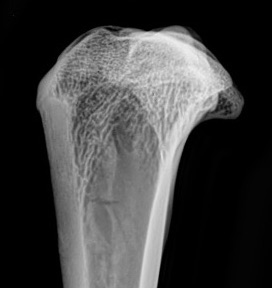


d

**Supplementary Figure 2.** (a) Image of craniocaudal radiograph showing the distal humeri of Dolly, (b) Image of craniocaudal radiograph showing the distal humeri of Morag, (c) Image of craniocaudal radiograph showing the proximal radii and ulnas of Dolly, and (d) Photographs of the actual bones shown in (c). The white arrows show osteophytes/areas of bone remodelling.

The radiographic scores for the humerus and radius/ulna for each limb were averaged to create the median score for the elbow joint. Dolly’s right elbow was scored as 2, and her left elbow as 3.


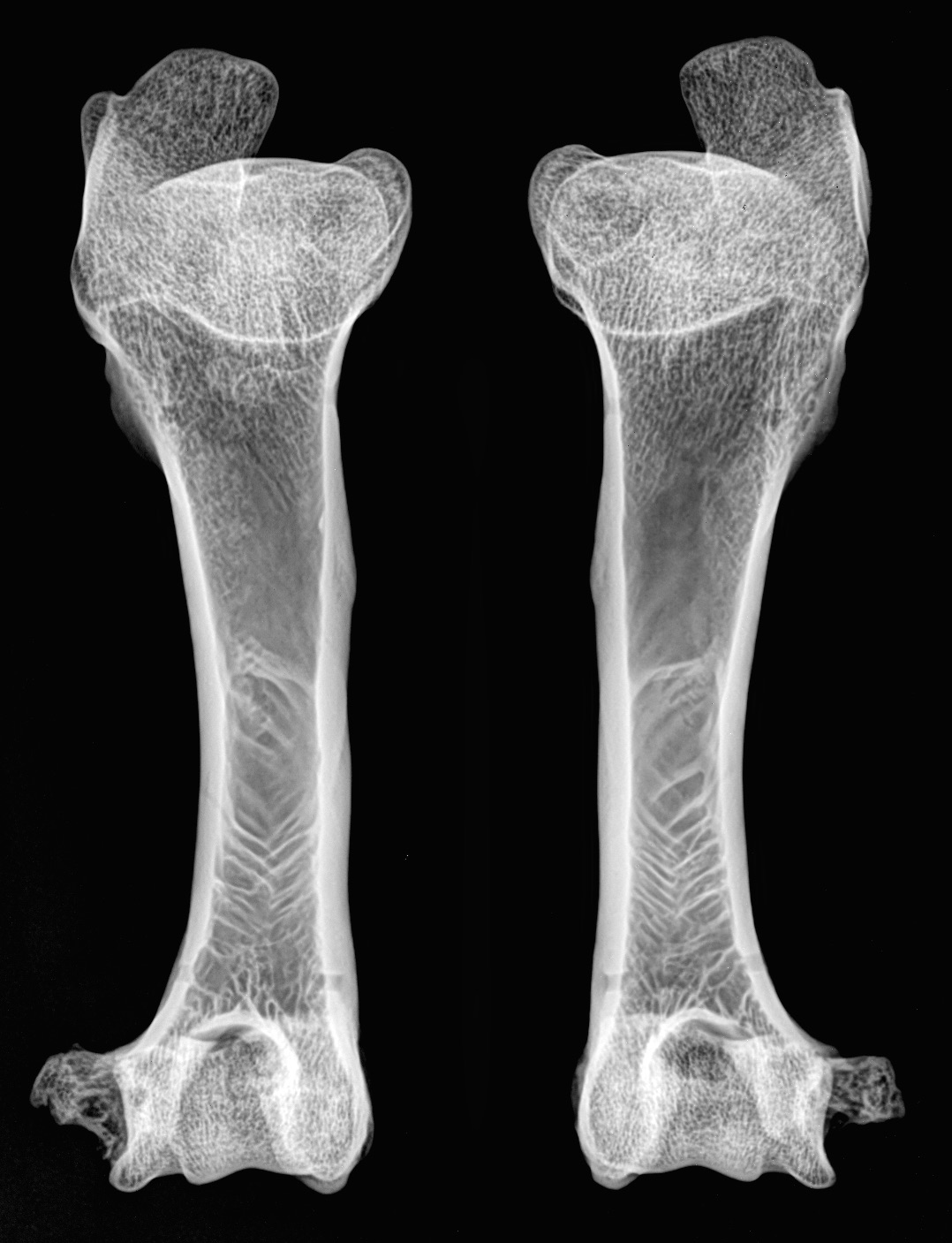


a

**R**

**L**


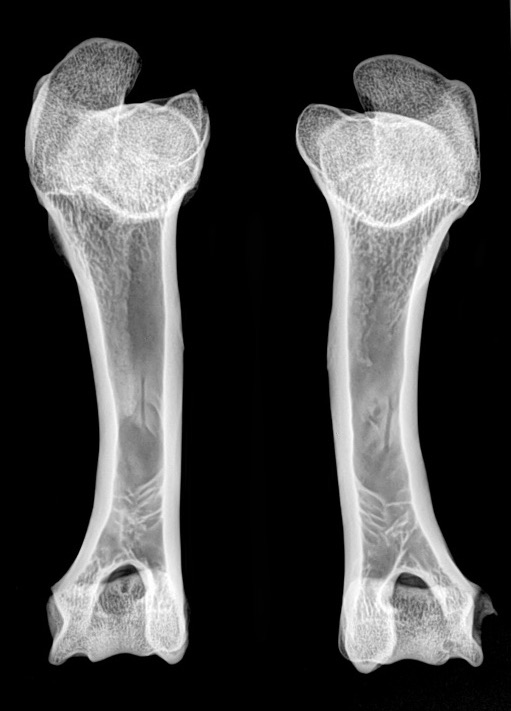


b

**R**

**L**


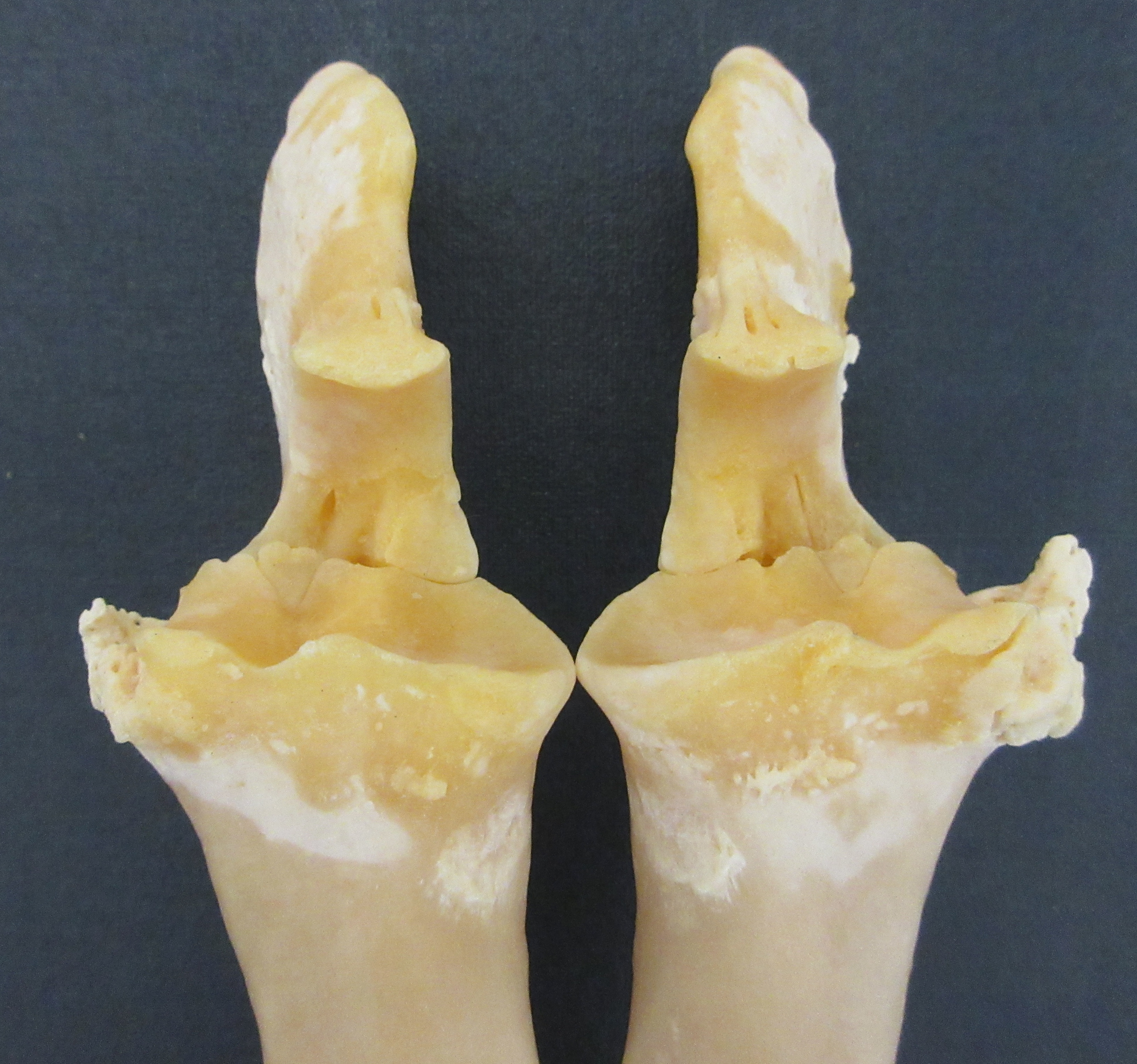


d

**R**

**L**


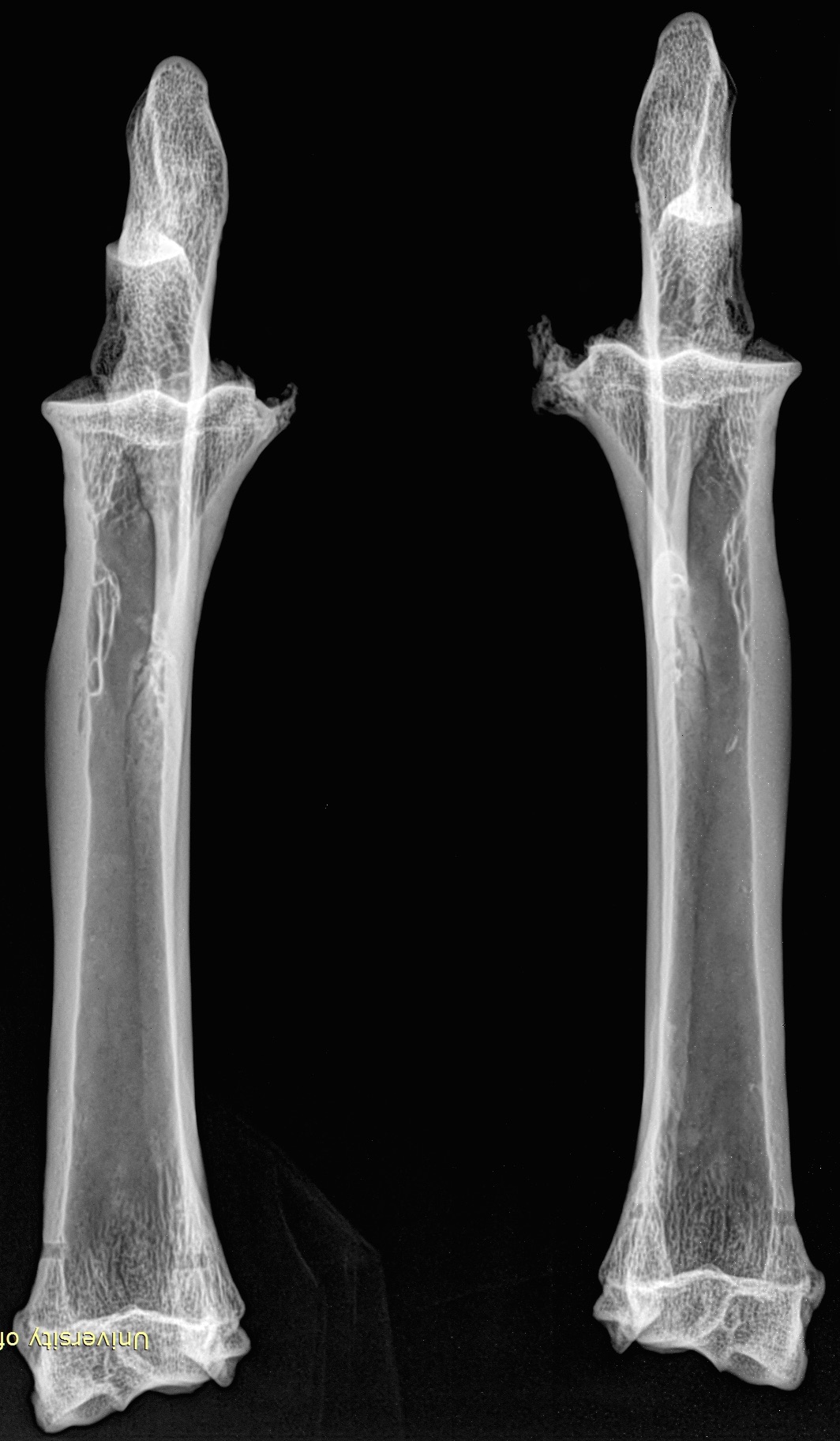

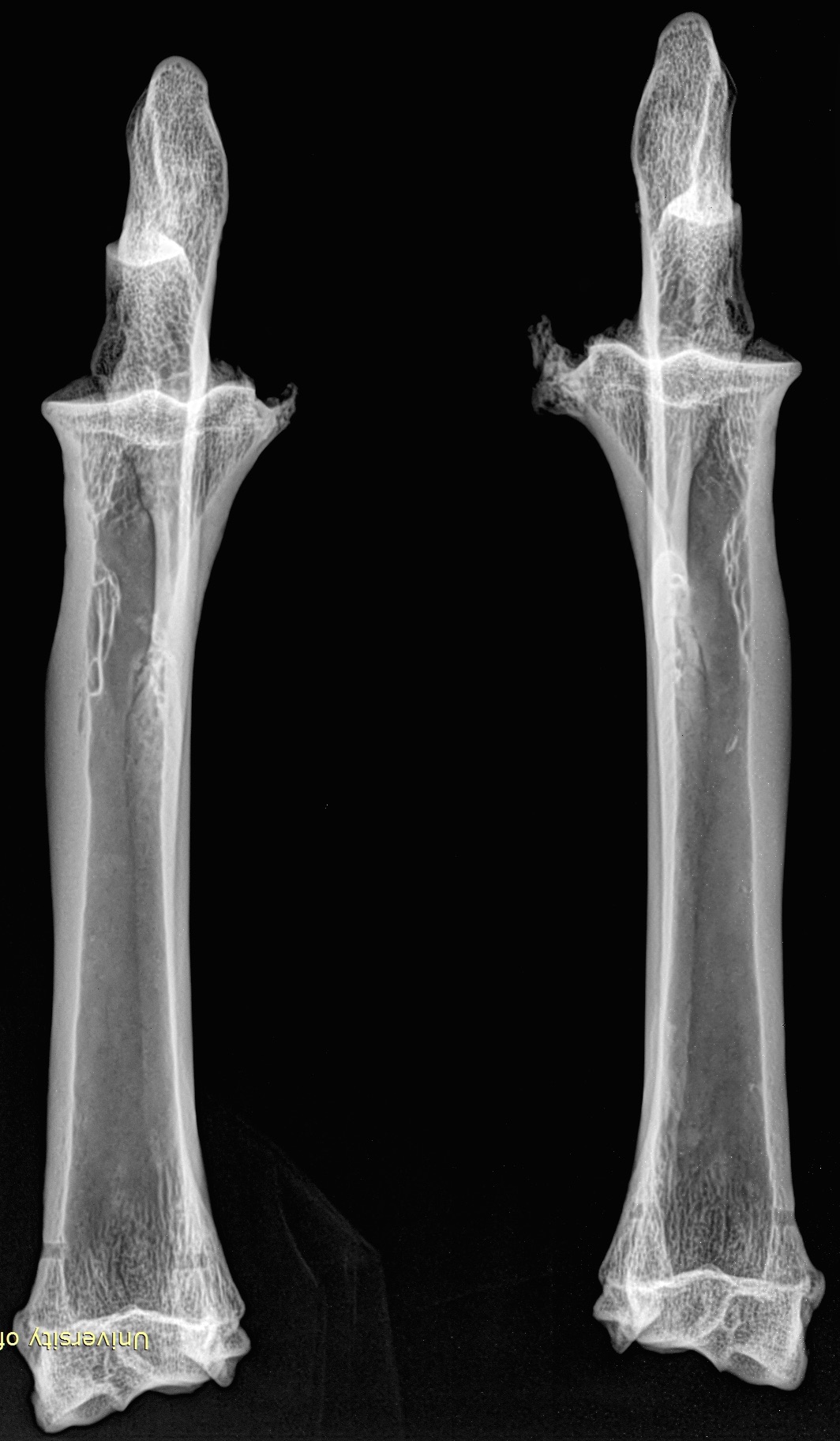


c

**L**

**R**

**Supplementary Table 1.** Background details on the four study animals.

| **Animal** | **Dolly** | **Bonnie** | **Megan** | **Morag** |
| --- | --- | --- | --- | --- |
| **Means of conception** | Nuclear transfer | Natural mating | Nuclear transfer | Nuclear transfer |
| **Nuclear Donor**  **(Genotype)** | Finn Dorset | Welsh Mountain x Finn Dorset | Welsh Mountain | Welsh Mountain |
| **Mitochondrial Donor (Genotype)** | Scottish Blackface | Scottish Blackface | Scottish Blackface | Scottish Blackface |
| **Born** | 5^th^ July 1996 | April 1998 | 19^th^ June 1995 | 22^nd^ June 1995 |
| **Died** | 14^th^ Feb 2003 | 20^th^ Mar 2008 | 15^th^ Jan 2009 | 27^th^ Mar 2000 |
| **Reason for euthanasia** | OPA | Age related | Age related | OPA |
| **Offspring** | 6 | 0 | 0 | 1 |

OPA: Ovine Pulmonary Adenocarcinoma (Jaagsiekte) – contagious, viral-induced tumour of the lungs

Bonnie: Dolly’s first lamb, conceived naturally to a Welsh Mountain ram.

Megan/Morag: Nuclear donor cells were from an embryo-derived epithelial cell line
